# High-fidelity simulation as a translational and educational platform for crisis ventilation: quantifying and mitigating tidal volume maldistribution during shared mechanical ventilation

**DOI:** 10.1007/s44186-026-00589-1

**Published:** 2026-10-03

**Authors:** Syed Husain, Aushja Syed, John Bockbrader, Tim Dunlea, Scott Winfield, Daniel Vazquez

**Affiliations:** 1 Department of Surgery, Ohio State University, N717 Doan Hall, 410 West 10th Ave, Columbus, OH 43210 USA; 2 Center for Design and Manufacturing Excellence, Ohio State University, 1314 Kinnear Road, Suite 1533, Columbus, OH 43212 USA; 3 Respiratory Therapy Department, Ohio State University, 294 Doan Hall 410 W. 10th Ave., Columbus, OH 43210 USA; 4 Medical Skill Development and Simulation Center, Ohio State University, 370 W. 9th Avenue, Columbus, OH 43210 USA; 5 Center for Research, Education, and Advancement of Transdisciplinary Exploration, Division of Trauma and Critical Care, Department of Surgery, Ohio State University, 1645 Neil Avenue, OH 43201 Columbus, USA

**Keywords:** Simulation, Split-ventilation, Tidal volume, Ventilator

## Abstract

**Background:**

Shared mechanical ventilation has been proposed during ventilator shortages, but unequal tidal volume delivery remains a major limitation when lung compliance differs. Professional societies have cautioned against this practice, yet the intervention has never been validated clinically, and ethical constraints preclude direct patient testing. Translational simulation—simulation applied beyond education to probe clinical systems, test crisis-driven innovations, and generate actionable safety data—offers a methodological pathway for evaluating interventions that cannot be studied in vivo [1, 2]. In parallel, high-fidelity simulation offers a controlled environment in which clinicians can be taught the physiologic hazards of shared ventilation without risk to patients.

**Methods:**

Two high-fidelity patient simulators were connected to one ventilator in volume-control assist-control mode using a parallel circuit. One simulator was set to normal compliance and the other to reduced compliance. Tidal volumes were measured before and after adjustment of the higher-flow limb with inline flowmeters. This study was designed as a translational simulation to evaluate a crisis-care equipment configuration under controlled, reproducible conditions. The objective was to use high-fidelity simulation as a controlled, reproducible experimental platform to (1) empirically quantify the compliance-driven tidal volume reduction predicted by respiratory-mechanics theory during shared ventilation, (2) test whether inline flowmeters with adjustable valves partially mitigate this, and (3) evaluate the feasibility of high-fidelity simulation as a translational and educational platform for crisis-care innovations that cannot ethically or logistically be studied in real patients. We hypothesized that (H1) shared ventilation of two lung units with mismatched compliance would produce clinically relevant tidal volume reduction in the low-compliance limb, and (H2) this would be counteracted by inline flowmeters with adjustable valves.

**Results:**

Without adjustment, the low-compliance simulator’s delivered volume fell markedly relative to its normal-compliance baseline; after flow regulation, it returned toward baseline at both 900 mL and 1100 mL tidal volume settings.

**Conclusions:**

This study demonstrates how high-fidelity simulation can function as a translational research platform for crisis-care innovations that are ethically and logistically impossible to study in clinical practice. Beyond confirming the physiologic limitations of unregulated ventilator sharing, the simulation generated reproducible, condition-specific tidal volume data that can inform crisis preparedness protocols and equipment pre-positioning decisions. This application of simulation as a systems-testing and device-evaluation tool, in addition to translating directly into a crisis-preparedness educational scenario, represents an underutilized but critically important role for simulation science in disaster and pandemic preparedness.

**Supplementary Information:**

The online version contains supplementary material available at https://doi.org/10.1007/s44186-026-00589-1.

## Introduction

Mechanical ventilators are complex medical devices designed to provide critical respiratory support. Mechanical ventilation is an invasive intervention with attendant risks and complications, including iatrogenic lung injury. Given their essential function, these devices have multiple built-in checks and alarms not only to ensure the delivery of safe, customizable respiratory support but also to minimize the risk of iatrogenic injury.

Epidemics of severe respiratory illness like the recent COVID pandemic led to a sudden surge in demand for mechanical ventilators, overwhelming baseline ventilator capacity in many resource-limited settings [3, 4]. As a result, clinicians across the country put forth proposals to address acute shortages of ventilators, including co-ventilating two patients by splitting a single ventilator using a parallel circuit [5–9]. While multiple publications describe the parallel circuit technique, it has never been validated clinically or through high-fidelity patient simulation. Reflecting concerns about uncontrolled tidal volume distribution, in 2020 the Society of Critical Care Medicine (SCCM), American Association for Respiratory Care (AARC), American Society of Anesthesiologists (ASA), Anesthesia Patient Safety Foundation (APSF), American Association of Critical-Care Nurses (AACN), and American College of Chest Physicians (CHEST) issued a joint statement cautioning against shared ventilation [10]. Yet this joint statement was based on theoretical reasoning and bench-level data rather than systematic testing in a physiologically representative environment.

This gap between theoretical concern and empirical evaluation highlights a broader challenge in crisis preparedness: how to rigorously test interventions that are ethically and logistically impossible to study in clinical practice. Translational simulation—defined as simulation integrated into healthcare systems to improve patient outcomes through means beyond direct education—offers a methodological solution [1, 2]. This approach has been used to test new clinical workspaces before opening, evaluate medical device configurations, reveal safety threats in care delivery systems, and prepare institutions for pandemic response [11–13]. Here we emphasize a systems-oriented simulation, where the simulation models the integrated ventilator–circuit–patient system together with the surrounding clinical and team environment, in contrast to an isolated skill or device, so that both investigators and learners can examine component interactions, failure modes, and decision-making under crisis conditions.

This article applies the translational simulation framework to a specific crisis-care challenge: shared mechanical ventilation. It makes two linked contributions. First, rather than using simulation solely to teach clinicians how to split a ventilator, we used high-fidelity simulation as a controlled experimental platform to (1) empirically quantify the reduction in tidal volume delivery to the low-compliance limb predicted by respiratory mechanics theory, and (2) test whether a pragmatic intervention—inline flowmeters with adjustable valves—can partially mitigate this reduction. Second, and central to the aims of this journal, this study demonstrates how a systems-oriented simulation environment can be converted into a reusable educational scenario that teaches surgical trainees and interprofessional crisis teams why shared ventilation is hazardous, how impedance mismatch alters tidal volume delivery, and what the limits of any mitigation strategy are—learning that cannot ethically be acquired at the bedside. Ultimately, we explore a replicable model for how simulation science can bridge the gap between theoretical feasibility and evidence-informed crisis decision-making.

## Methods

### Concept and design

**Fig. 1:**
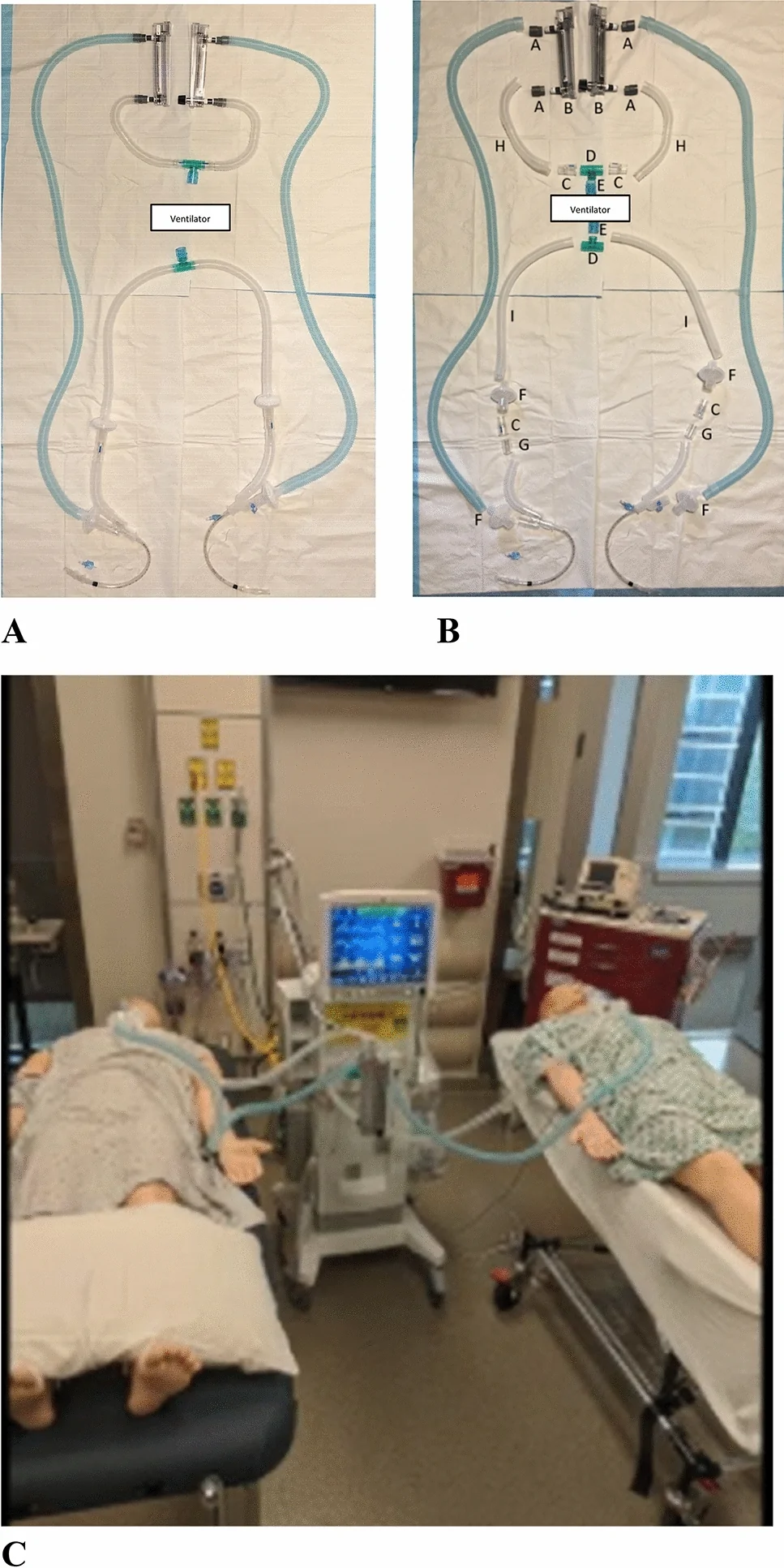
Simulation Setup. **A** Simplified. **B** Simplified – Deconstructed. **C** Screengrab from video of split ventilator setup. A. 3D printed Connectors X 4, B. Flowmeters X 2 (RMB-52-SSV and RMB-52, Dwyer®), C. One-way valves X 4 (One-way Valve, HUDSON RCI®), D. T connectors X 2 (Aerosol Tee Adaptor, Anti-spill, HUDSON RCI®), E. Couplers X 2 (Cuff Connector, AirLife™), F. HEPA filter X 4 (Guardian Disposable Bacterial / Viral Filter, Ventilab™), G. Couplers X 2 (Multi Adapter, HUDSON RCI®), H. Inspiratory limb, I. Expiratory limb

Two parallel respiratory circuits were created utilizing commonly used, commercially available connectors and ventilator tubing. In addition, commercially available flowmeters with and without flow‑control valves were used to measure and modulate flow to each patient. Figure 1B lists the equipment used for this simulation. The adapters used to attach the flowmeters to ventilator tubing were the only components not commercially available; these were 3‑D printed for the simulation. However, similar off‑the‑shelf adapters are commercially available in many markets. This is an NIH-funded study. We hypothesized that (H1) shared ventilation of two lung units with mismatched compliance would produce clinically meaningful tidal volume reduction in the low compliance limb, and (H2) this would be counteracted by inline flowmeters with adjustable valves.

### Construct validity

To ensure the model represents the mechanisms underlying the joint society statement, one simulator was programmed to normal compliance and the other to markedly reduced compliance (33%), reproducing the impedance mismatch that, in a parallel circuit, drives flow preferentially toward the more compliant lung. This design directly operationalizes the mechanism—uncontrollable, compliance-dependent partitioning of tidal volume—cited by the societies as the basis for advising against shared ventilation [10, 16, 17].

### Setup and equipment

**Fig. 2:**
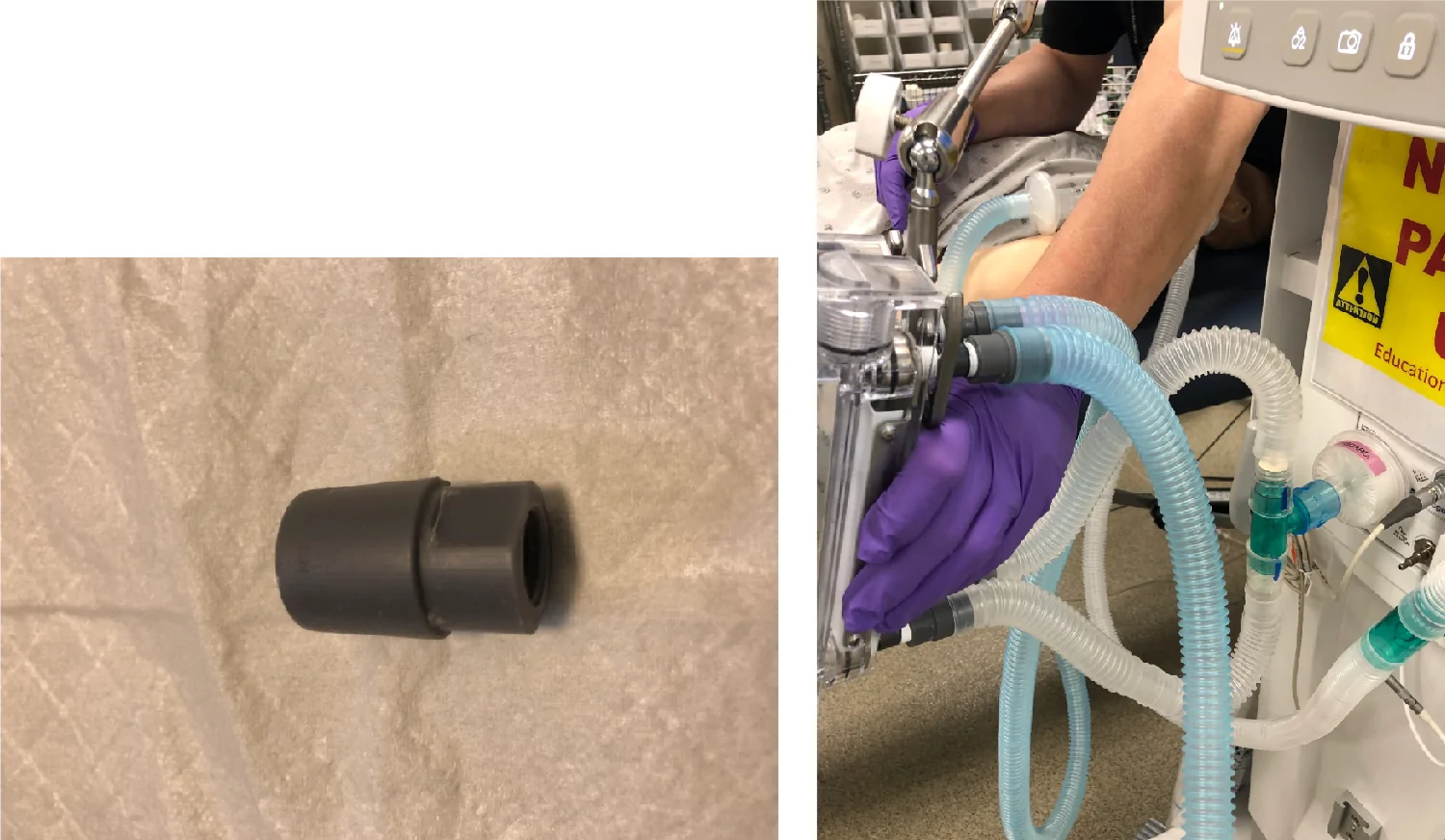
3-D printed connector

The circuit was assembled as follows (Fig. 1A and B):

- a. The inspiratory and expiratory ports of the ventilator are split using T‑connectors (Aerosol Tee Adaptor, Anti‑spill, HUDSON RCI®) to serve two patients.
- b. HEPA (High‑Efficiency Particulate Air) filters are placed on inspiratory and expiratory circuits for each patient (Guardian Disposable Bacterial/Viral Filter, Ventilab™).
- c. One‑way valves (One‑way Valve, HUDSON RCI®) are placed as indicated in the diagram to minimize backflow of air and cross‑infection between patients (Fig. 1). One‑way valves are important to prevent retrograde flow of air via the expiratory circuit from the patient with less compliant lungs to the patient with more compliant lungs.
- d. A flowmeter (RMB‑52‑SSV and RMB‑52, Dwyer®), with or without an adjustable valve, is attached to the inspiratory tubing of each patient. For co‑venting two patients, we recommend flowmeters with a 5–50 SCFH (standard cubic feet per hour) range. To ensure proper function, the flowmeters must remain in an upright position. Two types of flowmeters were tested in this simulation (with and without built‑in valves). In clinical use, both flowmeters should have adjustable valves and be of the same model and manufacturer to assure comparable calibration.
- e. 3‑D‑printed adapters were used to upsize the ¼‑inch NPT (National Pipe Thread) inlet/outlet of the flowmeter to 22‑mm internal diameter ventilator tubing (Fig. 2). The inlet/outlet dimensions may differ depending on the brand of flowmeter used.
- f. Two types of couplers (Multi Adapter, HUDSON RCI® and Cuff Connector, AirLife™) were used to connect T‑connectors to the ventilator ports and one‑way valves to ventilator tubing.

### Simulation

**Fig. 3:**
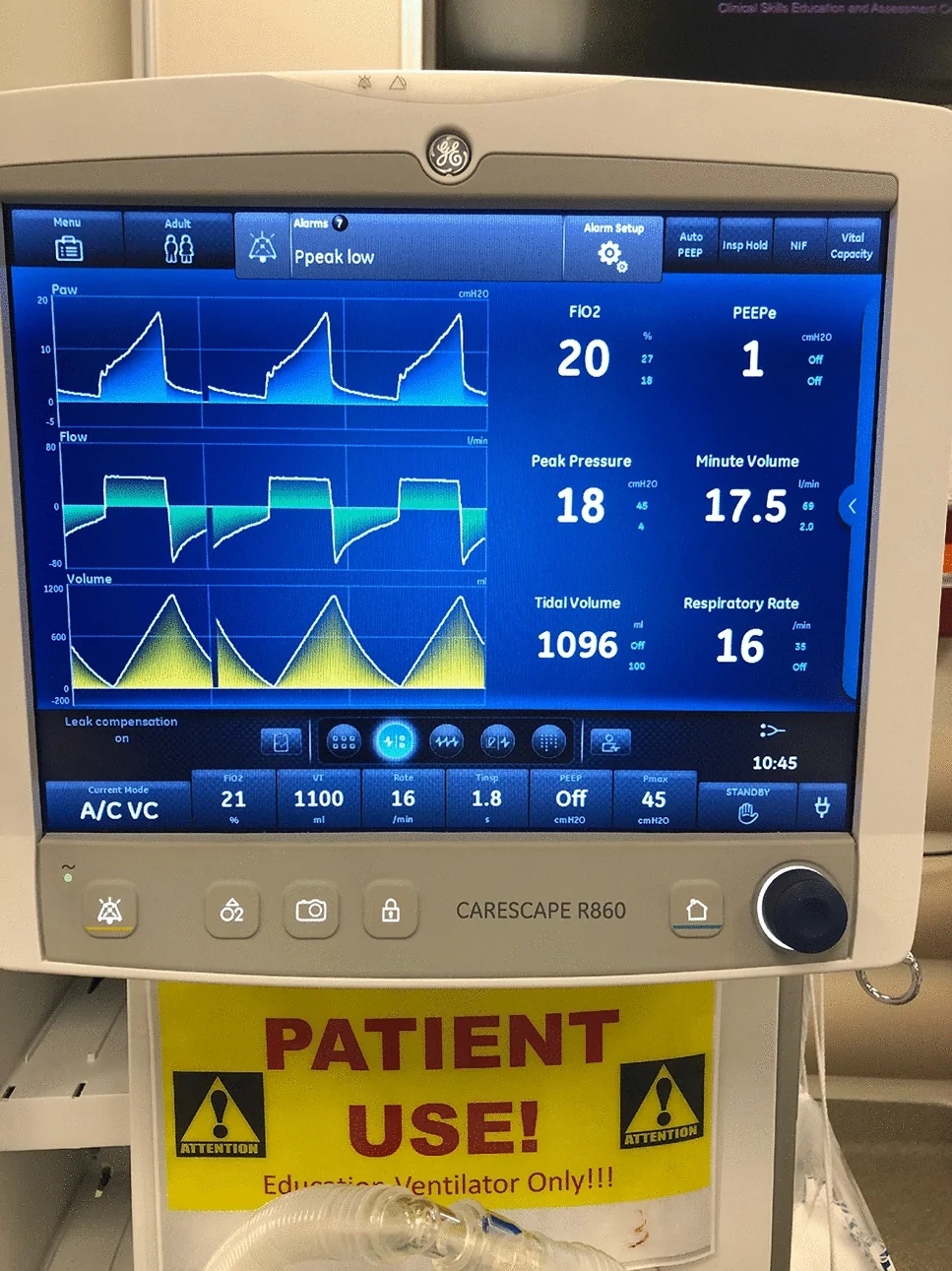
Ventilator settings

The following operational protocol was used in the high‑fidelity simulation (refer to Video 1):

- a. The ventilator was set to volume‑control in assist‑control mode.
- b. Two patient simulators (Laerdal SimMan® 3G PLUS) were attached to the ventilator using the described parallel circuit. Lung compliance was configured at a lower level (33%) on one patient simulator, while the other simulator was set at normal lung compliance.
- c. Usual, customary settings for FiO_2_, PEEP, and respiratory rate were used to initiate ventilation. The simulation was run on two tidal settings: 900 mL and 1100 mL. Ventilator settings used during the simulation are depicted in Fig. 3.
- d. Tidal volume delivered was measured at baseline and again after gradually closing the valve on the higher‑flow circuit until the floats in both flowmeters indicated comparable inspiratory flow rates. (Video 1).

### Prototype development and testing logistics

Prototype development was supported by a research voucher from the Center for Clinical and Translational Science (CCTS) at The Ohio State University (NIH grant UL1TR002733). Initial flowmeter selection and 3‑D printing of the flowmeter adapters were performed at the Center for Design and Manufacturing Excellence (CDME). Flowmeters were first tested in the laboratory using regulated air to ensure consistent measurement of air volume for each cycle under varying upstream pressures and downstream compliances.

Once lab testing confirmed acceptable performance, the prototype was tested in the Clinical Skills Education and Assessment Center (CSEAC), a state‑of‑the‑art, high‑fidelity training center capable of simulating actual patient‑care scenarios, including measurement of pulmonary compliance and tidal volumes. The deliberate progression from bench testing to high-fidelity simulation mirrors the translational research continuum, in which innovations advance through increasingly realistic testing environments before clinical application [14, 15].

### Data collection

**Fig. 4:**
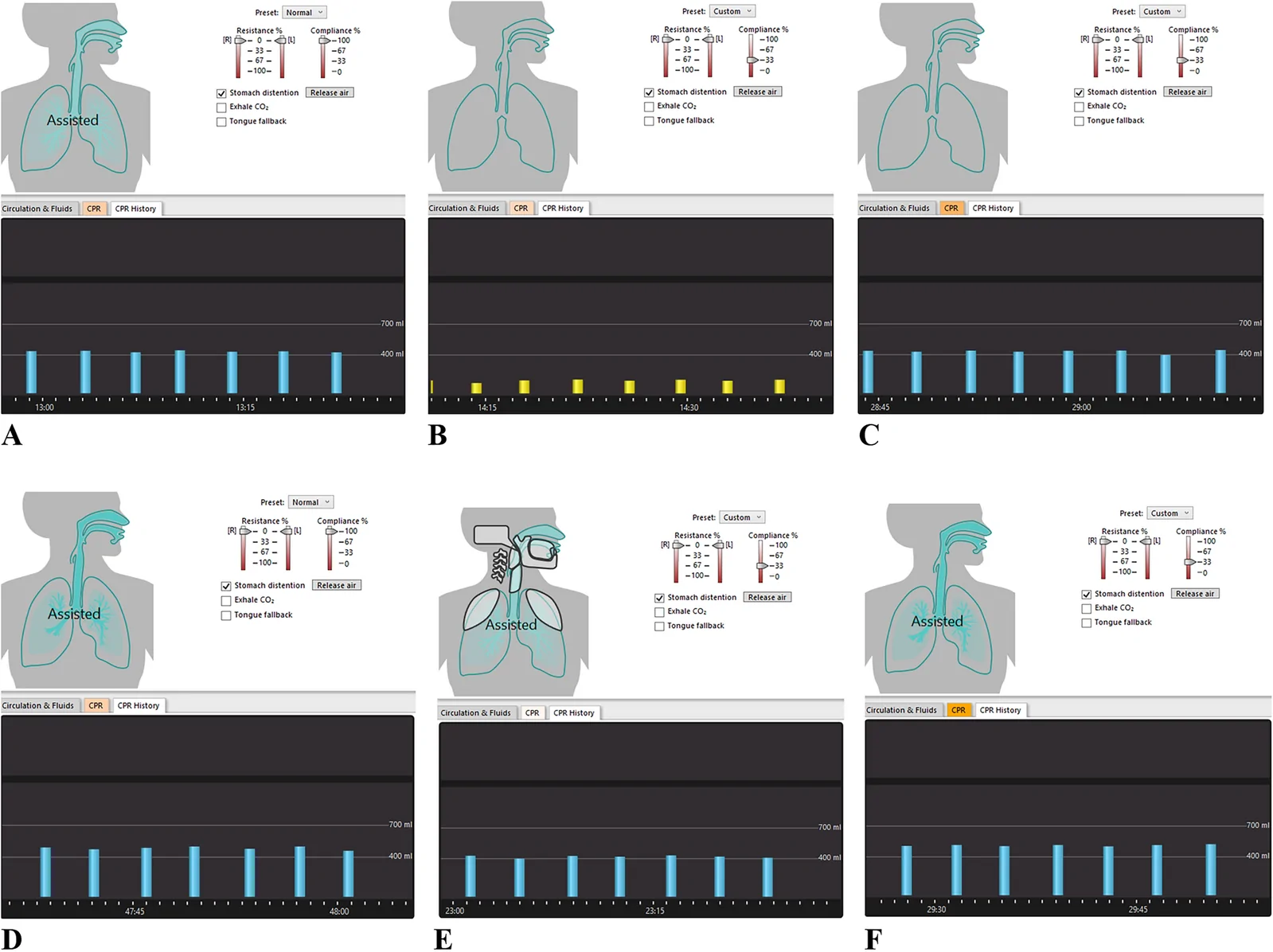
Split ventilator simulation. Tidal volume of 900 mL delivered to **A**. normal compliance (baseline) and low-compliance simulation **B**. before flow adjustment and **C**. after flow adjustment. **C**. Tidal volume of 1100 mL delivered to **D**. normal compliance (baseline) and low-compliance simulation **E**. before flow adjustment and **F**. after flow adjustment

The simulation was performed using two SimMan® 3G Plus simulation systems connected in parallel to a single ventilator circuit. Initial baseline measurements were obtained with normal lung compliance (100%). Simulations were conducted at ventilator tidal volume settings of 900 mL and 1100 mL. Delivered tidal volume was recorded from the simulator display monitor and served as the baseline value. Next, the simulations were repeated with one simulator set to reduced lung compliance (33%). Delivered tidal volumes were recorded before and after adjustment of the flowmeters to achieve equivalent flow through both limbs of the circuit. Following each change in ventilator or flowmeter settings, the system was allowed to equilibrate, as indicated by visibly stable plateaus over several breaths, before measurements were obtained. For each simulation condition, a maximum of seven consecutive tidal volume measurements were recorded to characterize measurement stability, and the mean value was used for analysis. Because the SimMan® output monitor displays tidal volume data graphically rather than numerically, graphical outputs were converted to numerical values using a publicly available graph digitization program. The converted values were subsequently used for analysis (Fig. 4).

### Data analysis

As a pilot, proof-of-concept design with a small number of measurements per condition, analyses were descriptive. For each of the six conditions (baseline, pre-adjustment, and post-adjustment at set tidal volumes of 900 mL and 1100 mL), the seven consecutive tidal volume measurements obtained from the digitized simulator output were summarized as mean ± standard deviation, range, and coefficient of variation (CV%). The CV% was used to characterize measurement stability and reproducibility within each condition. To quantify the effect of reduced compliance on delivered tidal volume, the change from the normal-compliance baseline to the reduced-compliance (pre-adjustment) condition was expressed as an absolute difference (mL) and a percent change. Similarly, the effect of flow adjustment was quantified as an absolute difference (mL) and a percent change.

### Feasibility outcome measures

Feasibility of the platform as a translational and educational tool was defined by three measures: (1) technical reproducibility—stable, repeatable readings across tidal volume delivery to the circuit; (2) discriminative capacity—the ability to demonstrate a difference in delivered tidal volume between the naïve and valve-regulated conditions; and (3) instructional usability—the completeness and adoptability of a facilitator-ready scenario (predefined learning objectives, facilitator guide, and structured debriefing) enabling other centers to deliver the scenario. Technical reproducibility and discriminative capacity are reported in the Results; the instructional scenario supporting the third measure is provided in the Supplementary Appendix.

## Results

As expected, the flowmeters indicated a much higher inspiratory flow rate for the model simulating normal lung compliance compared with the lower-compliance model. Simulator tidal volume readings were consistent with flowmeter observations. At a set total tidal volume of 900 mL, when at normal compliance, the simulator received approximately 436 mL at baseline. When at low compliance (33%), the simulator received only 141 mL before flowmeter adjustment; after adjustment, the delivered volume increased to approximately 431 mL. At a set total tidal volume of 1100 mL, when at normal compliance, the simulator received 487 mL at baseline. When at low compliance (33%), the simulator received 414 mL before flowmeter adjustment; after adjustment, the delivered volume increased to 501 mL (Table 1).

**Table 1:** Tidal volume delivered. “Pre” denotes before flow adjustment, indicating unequal inspiratory flow rates. “Post” denotes after flow adjustment, indicating equal inspiratory flow rates

	Measured TV (mL)	Resistance (%)	Compliance (%)
Set total TV: 900 mL			
Baseline	436	0	100
Pre	141	0	33
Post	431	0	33
Set total TV: 1100 mL			
Baseline	487	0	100
Pre	414	0	33
Post	501	0	33

In summary, at a tidal volume of 900 mL, the delivered tidal volume decreased by 295 mL or 68% when compliance was reduced, then recovered by 290 mL or 99% of baseline after flowmeter adjustment. At a tidal volume of 1100 mL, the delivered tidal volume decreased by 73 mL or 15% when compliance was reduced, then rebounded by 87 mL or 21% after flowmeter adjustment (slightly exceeding the normal compliance baseline, consistent with manual titration to matched float position rather than to a fixed target).

Across all six conditions, delivered tidal volume readings were highly stable within each condition, with coefficients of variation (CV) below 9% (900 mL setting: Baseline 436 ± 8 mL [range 427–448, CV 1.8%], Pre-adjustment 141 ± 12 mL [range 118–159, CV 8.9%], Post-adjustment 431 ± 16 mL [range 399–441, CV 3.6%]; 1100 mL setting: Baseline 487 ± 16 mL [range 462–504, CV 3.3%], Pre-adjustment 414 ± 11 mL [range 395–423, CV 2.7%], Post-adjustment 501 ± 7 mL [range 490–511, CV 1.4%]), confirming the reproducibility of measurements within each simulated configuration.

These findings empirically confirm the theoretical predictions underlying the 2020 joint society statement cautioning against ventilator sharing, providing among the first high-fidelity simulation data quantifying the magnitude of compliance-driven tidal volume reduction and its correction under controlled conditions [10].

### Feasibility outcomes

Feasibility of the platform was supported across all three prespecified measures. First, technical reproducibility was demonstrated by the low within-condition variability of delivered tidal volume across the seven consecutive breaths in each condition, with coefficients of variation below 9% in all six conditions (range 1.4–8.9%), indicating stable, repeatable readings once the circuit was assembled and equilibrated. Second, discriminative capacity was demonstrated by the platform’s ability to show clear, direction-consistent differences in delivered tidal volume between conditions: at the 900 mL setting, delivered volume in the low-compliance limb fell from a normal-compliance baseline of 436–141 mL and recovered to 431 mL after valve regulation, with an analogous pattern at 1100 mL (487 to 414 to 501 mL). The magnitude of these differences relative to the small within-condition variability confirms that the platform can distinguish the naïve from the valve-regulated state. Third, instructional usability was assessed by whether the scenario’s predefined learning objectives (Supplementary Appendix, Sect. "Methods") could be completed during facilitated use. The complete scenario, facilitator guide, and structured debriefing framework required to meet these objectives are provided in the Supplementary Appendix.

## Discussion

### Translational simulation as a systems-testing platform

The primary contribution of this work is methodological rather than purely technical. While the physiologic principles underlying tidal volume maldistribution in parallel circuits are well established, and while the joint society statement appropriately cautioned against unregulated ventilator sharing, neither the magnitude of the problem nor the potential for mitigation had been empirically evaluated in a physiologically representative environment [10, 16, 17]. High-fidelity simulation provided the controlled, reproducible, and ethically permissible platform necessary for this evaluation.

This application of simulation aligns with the emerging concept of translational simulation, which extends the role of simulation beyond education to directly examine clinical processes, test system-level interventions, and generate actionable safety data [1, 2]. Translational simulation has been used to test new clinical workspaces before opening, evaluate the integration of new technologies, perform prospective root cause analyses, and prepare institutions for pandemic response. The present study extends this paradigm to crisis-care device evaluation—using simulation not just to train clinicians, but also to generate empirical data about an equipment configuration that could not otherwise be tested [11–13].

This distinction is important for the simulation community. A 2021 survey of acute care hospitals found that only 15% used simulation for research, 37% used it for testing, and 30% used it for error analysis; the majority, 96%, used simulation for education [18]. The present study illustrates how simulation can serve as a rigorous investigative tool—functioning as a "clinical laboratory" for crisis-driven innovations that occupy the gap between theoretical feasibility and clinical implementation.

### From systems-testing platform to simulation curriculum

Although the circuit was developed as a translational systems-testing platform, it is directly translatable into a simulation curriculum for surgical residents, critical-care fellows, and interprofessional ICU teams who may be deployed under crisis standards of care. Within a mastery-learning framework, learners are tasked with (1) recognizing tidal volume maldistribution between two mismatched simulated patients, (2) titrating inline flowmeter valves to individualize delivered volume, and (3) identifying the failure modes—unilateral hypoventilation, volutrauma of the more compliant lung, and loss of independent PEEP/FiO₂ control—that underlie the 2020 joint society statement against shared ventilation [10]. Beyond technical titration, the scenario develops the nontechnical competencies emphasized in surgical simulation education: crisis resource management, closed-loop team communication, and ethically grounded triage under resource scarcity. Explicit learning objectives, a facilitator guide, and a structured debriefing framework are provided in the Supplementary Appendix so that the scenario can be adopted at other high-fidelity centers. Framed this way, the exercise teaches trainees not how to routinely split a ventilator, but why guideline bodies advise against it—by allowing them to observe the maldistribution, and the incompleteness of its mitigation, in a fully controlled environment. This use of simulation is consistent with established evidence for simulation-based mastery learning with translational outcomes and for simulation as a tool for prospective safety and systems-based practice [12, 13, 15].

### Implications for crisis preparedness

The simulation findings carry direct implications for crisis preparedness planning. First, the data empirically validate the concerns raised in the 2020 joint society statement: at a set tidal volume of 900 mL, the low-compliance simulator received only 141 mL—a volume incompatible with meaningful ventilation—instead of the 436 mL it received at normal compliance. This quantification moves the discussion from theoretical caution to measurable risk, providing crisis planners with concrete data to inform triage protocols and resource allocation decisions.

Second, the partial success of inline flowmeters in redistributing tidal volume suggests that if ventilator sharing were ever deemed necessary as a last-resort crisis measure, it need not be entirely uncontrolled. After flow adjustment, the low-compliance simulator’s tidal volume increased from 141 to 431 mL at the 900 mL setting and from 414 to 501 mL at the 1100 mL setting. While these volumes represent an important improvement toward individualized ventilation, they do not achieve fully independent control and could still inform, rather than establish, crisis standards of care.

Third, the reproducibility of the simulation protocol itself has value for crisis preparedness. The equipment list, circuit configuration, and testing methodology described here can be replicated at any institution with high-fidelity simulation capabilities, enabling local systems testing and protocol development before a crisis occurs. This proactive, simulation-based approach to crisis preparedness—testing equipment configurations, identifying failure modes, and developing mitigation strategies in advance—exemplifies the systems-testing function that simulation is uniquely positioned to serve [12, 13].

### Physiologic considerations

Several groups described simple ventilator‑splitting arrangements using Y‑connectors placed on the ventilator ports [9, 19–22]. Although these systems appear straightforward, they neglect basic principles of respiratory system mechanics: in a parallel circuit, flow preferentially follows the path of least resistance, so patients with higher lung compliance receive a disproportionately larger share of the delivered tidal volume, whereas those with stiffer lungs may receive very little [23]. This difference makes it impossible to reliably determine or control patient‑level tidal volumes—a parameter central to lung‑protective ventilation—and exposes the more compliant lung to volutrauma and barotrauma while leaving the less compliant lung underventilated, with risks of atelectasis and worsening hypoxemia [23, 24]. Reflecting these concerns with shared ventilation, in 2020 the Society of Critical Care Medicine (SCCM), American Association for Respiratory Care (AARC), American Society of Anesthesiologists (ASA), Anesthesia Patient Safety Foundation (APSF), American Association of Critical-Care Nurses (AACN), and American College of Chest Physicians (CHEST) issued a joint statement cautioning against it with the reasoning that most of the tidal volume delivered by the ventilator will go to the more compliant patient [10]. Pressure‑controlled modes may limit the risk of barotrauma but do not overcome the fundamental problem of unequal volume distribution when compliances diverge, meaning that the stiffest lung can still be severely under‑ventilated despite apparently “safe” ventilator pressures [16, 17, 23, 24].

Our simulation empirically confirmed these theoretical predictions and demonstrated that incorporating inline flowmeters with adjustable valves into each circuit enables deliberate redistribution of inspiratory flow. Progressive restriction of the higher-flow limb resulted in convergence of flowmeter readings, despite persistent differences in lung compliance. This effect was reproducible across both tested tidal volume settings.

## Limitations and future directions

This is a pilot proof-of-concept study with a limited number of experimental conditions and measurements obtained from a single circuit assembly. The findings are hypothesis-generating; larger studies spanning a wider range of compliance/resistance combinations, additional ventilation modes, dynamic changes in lung mechanics, and longer durations are needed before conclusions regarding safety or educational effectiveness can be generalized.

Flowmeters do not directly measure tidal volume. Their use provided a practical surrogate that enabled observation and dynamic adjustment of inspiratory flow. The experimental setup does not capture the physiologic instability, spontaneous respiratory efforts, secretion burden, or rapidly evolving lung mechanics typical of critically ill patients. Additionally, the system does not allow independent adjustment of FiO_2_, PEEP, respiratory rate, or inspiratory time, and therefore cannot replicate individualized ventilator management.

These limitations, however, also define the future research agenda for simulation-based crisis preparedness. Subsequent studies could incorporate dynamic compliance changes during the simulation to model clinical deterioration, test the system with more than two simulators, evaluate the training requirements for bedside clinicians to manage flow adjustment in real time, and assess whether in situ simulation of the complete workflow—including team communication, monitoring, and troubleshooting—reveals latent safety threats not apparent in the current bench-to-simulator design. Such iterative, simulation-based testing represents the systems-probing function that translational simulation is designed to serve [1, 2, 14].

## Conclusion

This study demonstrates that high-fidelity simulation can function as a translational research platform for crisis-care innovations that are ethically and logistically impossible to study in clinical practice. Beyond confirming the physiologic limitations of unregulated ventilator sharing and providing proof of concept for a flow-regulation strategy, this work illustrates a broader methodological principle: simulation science has an underutilized but critically important role as a systems-testing and device-evaluation tool for disaster and pandemic preparedness. Moreover, the same pilot-tested platform is directly translatable into a structured educational scenario that teaches surgical trainees and interprofessional teams the physiology, hazards, and ethical dimensions of shared ventilation. As simulation programs increasingly pursue translational goals—applying simulation not only for learner development but for prospective safety evaluation, device and workflow testing, and institutional readiness assessment—crisis-driven innovations like shared ventilation represent a natural and high-impact domain for this evolving discipline [1, 2, 13].

## Supplementary Information

Below is the link to the electronic supplementary material.

Supplementary file1 (DOCX 18 kb)

## Data Availability

The data that support the findings of this study are available from the corresponding author upon reasonable request.
