# Supplementary material for: High-fidelity simulation as a translational and educational platform for crisis ventilation: quantifying and mitigating tidal volume maldistribution during shared mechanical ventilation
Source: Global Surg Educ. 2026 Oct 3;5(1):185. doi: 10.1007/s44186-026-00589-1 (PMC13633965; doi:10.1007/s44186-026-00589-1)
Supplement: Supplementary file 1 [file 44186_2026_589_MOESM1_ESM.docx]

**Supplementary Appendix**

**Learning Objectives, Facilitator Guide, and Structured Debriefing Framework for a High-Fidelity Shared-Ventilation Crisis Scenario**

This appendix accompanies the parallel-circuit shared-ventilation platform described in the main text. It is designed to allow other high-fidelity simulation centers to reproduce the scenario for surgical residents, critical-care fellows, and interprofessional ICU teams who may be deployed under crisis standards of care. The scenario is intended to teach why guideline bodies advise against shared ventilation, not to establish it as a routine practice.

## **Section 1. Target Learners and Prerequisites**

## **Primary audience:** Surgical residents, critical-care and anesthesiology fellows, respiratory therapists, and critical-care nurses.

## **Recommended group size:** 4–6 learners per run, ideally interprofessional.

## **Prerequisite knowledge:**

## Basic modes of mechanical ventilation (volume-control and pressure-control, assist-control).

## The relationship between compliance, resistance, and delivered tidal volume.

## Familiarity with lung-protective ventilation targets.

## **Faculty required:** One content-expert facilitator (intensivist, anesthesiologist, or pulmonologist), one simulation technician, and one respiratory therapist or biomedical engineer familiar with the circuit.

## **Section 2. Explicit Learning Objectives**

## By the end of the scenario, learners will be able to:

## **Cognitive (knowledge) objectives**

## 1. Explain how impedance (compliance) mismatch drives preferential flow toward the more compliant lung in a parallel shared circuit.

## 2. Predict the direction and approximate magnitude of tidal volume maldistribution when two mismatched patients share one ventilator.

## 3. State the specific hazards that underlie the 2020 joint society statement against shared ventilation: unilateral hypoventilation of the stiffer lung, volutrauma/barotrauma of the more compliant lung, and loss of independent PEEP, FiO₂, respiratory rate, and inspiratory-time control (Society of Critical Care Medicine et al., 2020).

## 4. Articulate why pressure-controlled modes do not resolve unequal volume distribution when compliances diverge.

## **Psychomotor (technical) objectives** 5. Correctly identify tidal volume maldistribution between two mismatched simulated patients using flowmeter and simulator readouts. 6. Titrate inline flowmeter valves to redistribute inspiratory flow and approximate individualized delivered volume. 7. Recognize the limits of flow regulation—demonstrating that maldistribution is mitigated but not eliminated and that independent ventilator parameters cannot be restored.

## **Behavioral (nontechnical) objectives** 8. Demonstrate crisis resource management, including role assignment and closed-loop communication during troubleshooting. 9. Apply an ethically grounded framework for triage and resource allocation under crisis standards of care. 10. Formulate and communicate a recommendation about whether shared ventilation is an acceptable last-resort option, citing observed data.

## **Section 3. Facilitator Guide**

## **3.1 Equipment checklist**

## Two high-fidelity patient simulators capable of independent compliance settings (e.g., Laerdal SimMan 3G PLUS).

## One ICU ventilator with volume-control assist-control mode.

## Parallel circuit components: two T-connectors, four one-way valves, four HEPA filters, two inline flowmeters with adjustable valves (5–50 SCFH range, matched model/manufacturer), couplers, and adapters (3-D printed or off-the-shelf) to connect flowmeters to 22-mm tubing.

## Monitor displays for delivered tidal volume on each simulator.

## **3.2 Simulator programming**

## Simulator A: normal lung compliance.

## Simulator B: markedly reduced compliance (set to ~33% of normal) to reproduce the impedance mismatch cited by the societies.

## Initial ventilator settings: usual FiO₂, PEEP, and respiratory rate; run the scenario at both 900 mL and 1100 mL set total tidal volume.

## **3.3 Scenario flow (approximately 45–60 minutes)**

## *Pre-briefing (10 minutes):* Orient learners to the simulators, circuit, monitors, and flowmeters. Establish psychological safety and a fiction contract. State that the goal is to understand shared-ventilation physiology, not to endorse the practice.

## *Phase 1 — Baseline recognition (10 minutes):* Learners initiate shared ventilation and observe delivered tidal volumes. Expected finding: the compliant simulator receives a disproportionately large share while the stiff simulator is severely underventilated (e.g., ~140 mL vs ~440 mL at the 900 mL setting). Prompt learners to interpret the maldistribution and its clinical consequences.

## *Phase 2 — Mitigation attempt (10 minutes):* Learners progressively close the valve on the higher-flow limb until flowmeter floats indicate comparable inspiratory flow. Expected finding: convergence of delivered volumes (e.g., stiff simulator rising toward ~430 mL at 900 mL). Prompt learners to recognize the improvement.

## *Phase 3 — Limits and failure modes (10 minutes):* Facilitator introduces or highlights that FiO₂, PEEP, rate, and inspiratory time remain shared and cannot be individualized, and optionally simulates deterioration (further compliance drop) to show instability. Learners articulate residual hazards.

## **3.4 Anticipated learner errors and facilitator cues**

## Failing to recognize maldistribution → cue learners to compare the two delivered-volume readouts directly.

## Assuming pressure-control resolves the problem → prompt reasoning about compliance-dependent volume partitioning.

## Over-crediting flow regulation as a definitive fix → highlight retained shared parameters and inability to set lung-protective targets per patient.

## Neglecting communication → assign explicit roles (airway/ventilator, flow titration, monitoring, team lead).

## **Section 4. Structured Debriefing Framework**

## A three-phase debriefing (reactions → analysis → summary) is recommended, using advocacy-inquiry to explore the reasoning behind learner actions.

## **4.1 Reactions phase (5 minutes)**

## Open-ended prompt: invite learners to describe their immediate impressions and emotional response to managing two mismatched patients on one ventilator.

## **4.2 Analysis phase (15–20 minutes)** — guided by objectives:

## *Physiology and recognition*

## What did the delivered-volume data show at baseline, and why did the compliant lung receive more?

## How did the flowmeter readings correspond to delivered tidal volumes?

## *Mitigation and its limits*

## What changed after valve titration, and what did not?

## Which ventilator parameters remained shared, and what are the clinical implications of being unable to individualize them?

## Would pressure-control have solved the problem? Why or why not?

## *Guideline and evidence linkage*

## How do the observed findings map onto the specific hazards named in the 2020 joint society statement (Society of Critical Care Medicine et al., 2020)?

## Given the data, is shared ventilation an acceptable last-resort strategy? Under what constraints, if any?

## *Teamwork and ethics*

## How were roles, communication, and decisions handled under crisis conditions?

## How should scarce ventilator resources be allocated when individualized control is impossible?

## **4.3 Summary phase (5 minutes)**

## Learners state two or three key takeaways in their own words.

## Facilitator reinforces the central message: shared ventilation produces clinically relevant, compliance-dependent maldistribution that inline flow regulation can reduce but not eliminate, and that independent lung-protective ventilation cannot be delivered to both patients simultaneously.

## **Section 5. Assessment and Feasibility Measures**

## Consistent with the feasibility outcomes reported in the main text, adopting centers may assess:

## **Technical reproducibility:** stability of maldistribution data across replicate runs

## **Discriminative capacity:** ability to detect a difference in delivered tidal volume between naïve and valve-regulated conditions.

## **Instructional usability:** completion of the predefined learning objectives during facilitated runs with structured debriefing.

## A simple checklist mapped to the psychomotor objectives (Section 2, items 5–7) can be used for formative, mastery-oriented assessment, consistent with simulation-based mastery learning approaches (McGaghie et al., Medical Education, 2014).

## **Section 6. Safety and Ethical Framing Statement for Facilitators**

## Facilitators must explicitly frame the scenario as an educational demonstration of hazard, not a validated clinical protocol. The takeaway for trainees is to understand why professional societies advise against shared ventilation and to appreciate the incompleteness of any mitigation, so that the practice is reserved—if ever—for the most extreme last-resort crisis conditions with full awareness of its limitations.
